# How Do the Abdominal Muscles Change during Hypopressive Exercise?

**DOI:** 10.3390/medicina57070702

**Published:** 2021-07-09

**Authors:** Iria Da Cuña-Carrera, Alejandra Alonso-Calvete, Mercedes Soto-González, Eva M. Lantarón-Caeiro

**Affiliations:** 1 Faculty of Physiotherapy, University of Vigo, 36310 Vigo, Spain; iriadc@uvigo.es (I.D.C.-C.); alejalonso@uvigo.es (A.A.-C.); evalantaron@uvigo.es (E.M.L.-C.); 2 REMOSS Research Group, Faculty of Education and Sports Science, University of Vigo, Pontevedra, 36310 Vigo, Spain

**Keywords:** ultrasound imaging, abdominal muscles, hypopressive exercises, back care

## Abstract

*Background and objective*: Prior studies have reported an activation of abdominal muscles during hypopressive exercises in women with pelvic floor disfunction. However, no previous research analyzed the effects of hypopressive exercise on abdominal muscles in healthy populations to understand the normal biomechanics of this area. The aim of this study was to examine the thickness of abdominal muscles at rest and during hypopressive exercise in supine and standing positions with ultrasound imaging in healthy adults. *Methods*: A cross-sectional study was carried out in 99 healthy university students. The thickness of the abdominal muscles at rest and during hypopressive exercise was assessed with ultrasound imaging in supine and standing positions. *Results*: During hypopressive exercise, there was a significant increase in the muscle thickness of transversus abdominis (*p* < 0.001) and internal oblique (*p* < 0.001) in supine and standing positions. External oblique only increased its thickness significantly in the standing position (*p* < 0.001) and rectus abdominis did not change during the hypopressive exercise in any position (*p* > 0.05). In conclusion, hypopressive exercises seem to increase the thickness of the deepest and most stabilized muscles such as transversus abdominis and internal oblique. *Conclusions*: These findings should be considered for future interventions with hypopressive exercises in healthy subjects.

## 1. Introduction

Hypopressive exercises (HE) are described as postural exercises which relax the diaphragm, decrease intra-abdominal pressure, and may activate abdominal and pelvic floor muscles (PFM) when performed [1,2]. The HE can be performed in different positions such as supine, seated or standing, and arms and legs can variate their positions through different movements too [1]. This performance in combination with apnea and pressure changes has been described as able to activate the postural muscles, such as the deep abdominal muscles and PFM [3]. For this reason, HE have been largely used in the treatment of PFM dysfunctions, such as urinary incontinence, and specially in postpartum women with benefits in the pelvic and abdominal area [1,2,3]. Nevertheless, there is still no consensus nowadays about the effects of HE on the abdominal wall. Prior research has found an activation of the abdominal muscles during HE, measured by surface electromyography [4,5]. However, this measuring method only provides information about superficial tissues, and other techniques such as ultrasound imaging have been described to precisely evaluate deep anatomical structures [6] and muscle dimensions [7,8] which could provide a more in-depth analysis of this area. Moreover, among the imaging modalities, ultrasound imaging has been shown as the most cost-effective and feasible method for measurements of the muscular tissue [6], since muscles can be visualized in real-time and measurements can be obtained while relaxed but also during movements, different states of contraction or different positions, such as during HE [6]. To the best of the authors’ knowledge, only one study has analyzed the effects of HE in abdominal muscles with ultrasound imaging, and it found an increase of the thickness of the transversus abdominis and the internal and external obliques when HE was performed by women with PFM disfunction [9]. However, there is no previous research analyzing the effects of HE on the abdominal walls of the healthy population, but its importance is remarkable since no previous investigations have described the changes in abdominal muscles during these exercises under non-pathological conditions, so there is still a lack of knowledge of the normal biomechanics of abdominal muscles during HE. Thus, according to previous considerations, the purpose of this study was to analyze the thickness of abdominal muscles with ultrasound imaging during rest and HE in supine and standing positions.

## 2. Materials and Methods

### 2.1. Design and Participants

A cross-sectional experimental study was carried out with the purpose of assessing the thickness of abdominal muscles at rest and during the HE in supine and standing positions.

Sample size was based on data from Amerijckx et al. [10] using the variable “transversus abdominis thickness (average) at the end of relaxed expiration” (4.1 ± 1.4) versus “transversus abdominis thickness (average) at the end of full expiration” (5.9 ± 2.1). G*Power software 3.1 (Heinrich-Heine-Universitat, Dusseldorf, Germany) was used with a power of 0.95 and an α error of 0.05, and a sample of 13 subjects was estimated.

All participants were healthy university students with no history of chronical diseases or contraindications for exercise. Inclusion criteria were male and nulliparous women and being able to perform HE correctly. Exclusion criteria were pregnancy, arterial hypertension and neurological or autoimmune tissue disorders, according to previous studies [5,11,12]. The initial sample size was 102 participants and after inclusion and exclusion criteria 99 participants were voluntarily recruited for this study.

All subjects received oral and written information about the study and signed a written informed consent. Ethical approval was granted by the Ethics Committee of the University of Vigo (code 20072018/44), and the principles of the Declaration of Helsinki were followed.

### 2.2. Hypopressive Exercises Training

All subjects were instructed by a qualified physical therapist on how to perform the HE correctly in a familiarization session before taking any measurements. Those participants who did not learn the exercises or did them wrong came to another instructed session. After the two sessions, and in accordance with the criteria of the qualified physical therapist, all subjects who did not learn the exercise correctly were excluded from the study (*n* = 3).

These HE training sessions consisted of several steps. First, subjects were asked to do a spine elongation with neutral pelvis and scapular muscle activation. Second, participants were asked to perform three normal breathing cycles followed by a slow deep exhalation in the last breath. Finally, after the exhalation, they were asked to hold a breath in with expansion and lift of the ribs [1,13]. All these instructions were followed by the participants during the training session and the physical therapist checked all the parameters in order to ensure the proper performance of the exercise. Moreover, this training was performed in supine and standing positions.

### 2.3. Procedures

The thickness of abdominal muscles was measured using 5–10 mHz lineal ultrasounds transduce (SonoSite M-Turbo^®^, Amsterdam, Netherlands). Previous research has studied muscle thickness with ultrasonography and has reported its reliability when measuring the musculoskeletal system [6,14]. The measurements were carried out by a physical therapist with knowledge of musculoskeletal ultrasound imaging and who was trained in measuring abdominal muscles’ thickness. For rectus abdominis, the probe was located on the anterior abdominal area and on the dominant side of the subject, just lateral to the navel [15]. For external oblique, internal oblique and transversus abdominis the probe was located laterally between the iliac crest and rib cage, also on the dominant side of the subject [5,11,12]. Once the muscle was located, the least pressure possible was applied and the location of the probe was marked on the skin of the subject so the thickness measurements were always recorded in the same place, using the on-screen Calipper provided by the ultrasonography equipment. This Calipper stayed perpendicular inside the hyper-echoic area in the muscle, between the fascial borders [15,16]. Among each exercise, 5 min of rest was taken in order to avoid mistakes in their performance caused by muscle fatigue [17]. All data were recorded with the subject in both supine and standing positions in order to subsequently check the differences in rectus abdominis and transversus abdominis and internal and external oblique.

### 2.4. Statistical Analyses

Statistical analysis was carried out using the statistical software R version 3.6.3 [18]. Descriptive data about subjects and the thickness of the muscles studied are presented as mean ± standard deviation (SD). Linear mixed models were fitted using the R package lme4 [19] in order to analyze the differences between abdominal muscles’ thickness in both positions, supine and standing, and measurements were taken during rest in the same way as during hypopressive exercise. The situation (rest or hypopressive exercise) and the position (supine or standing) were fitted as fixed effects, and subjects were modelled as random effects to account for the repeated measurements.

The assumptions of homogeneity and normal distribution of the residuals were verified for each model, without revealing specific problems. Pair-wise comparisons for the interaction between the variables situation and position were conducted using the R package emmeans [20].

Effect size (ES) was calculated using Cohen’s d Pl [21] and classified as trivial (d < 0.2), small (0.2 ≤ d ≤ 0.5), medium (0.5 < d ≤ 0.8) or large (d > 0.8). For all analyses, the significance level was set at *p* < 0.05.

## 3. Results

In this study, 99 healthy subjects (64% women; 36% men; age 22.43 ± 3.56 years; body mass index 22.81 ± 2.75) were recruited, with the aim of analyzing the effects of HE on abdominal thickness. The main results after statistical analysis are detailed in Table 1, comparing the thickness of each muscle (transversus abdominis, internal oblique, external oblique and rectus abdominis) in both rest and hypopressive situations and also in both supine and standing positions. Interactions between situation and position are also shown.

As shown in Table 1, transversus abdominis increased its thickness significantly during HE in comparison with rest, both in the supine (*p* < 0.001; d = 1.0839, large) and standing positions (*p* < 0.001; d = 1.0494, large). Transversus abdominis’ thickness is significantly higher in standing position in comparison with supine, both in rest (*p* < 0.001; d = 1.1429, large) and HE (*p* < 0.001; d = 1.1084, large).

Internal oblique increased its thickness significantly during HE in comparison with rest, both in supine (*p* < 0.001; d = 0.8157, large) and standing position (*p* < 0.001; d = 0.7543, medium). There were no significant differences in thickness according to the position neither in rest (*p* = 0.3205; d = 0.2031, small) nor in HE (*p* = 0.7632; d = 0.0431, trivial).

External oblique increased its thickness significantly during HE in comparison with rest only in the standing position (*p* < 0.001; d = 0.6449, medium). There were no significant differences between rest and HE in supine position (*p* = 0.0922; d = 0.2413, small), or between both positions neither in rest (*p* = 0.2; d = 0.1814, trivial) nor in HE (*p* = 0.12; d = 0.2217, small).

Rectus abdominis had no significant differences in its thickness during HE in comparison with rest, neither in supine position (*p* = 0.3228; d = 0.1414, trivial) nor in standing (*p* = 0.9534; d = 0.0083, trivial). There were also no significant differences according to the position neither in rest (*p* = 0.2110; d = 0.1791, trivial) nor in HE (*p* = 0.7492; d = 0.0457, trivial).

## 4. Discussion

The results of this study suggest that HE is able to significantly increase the thickness of the deep abdominal muscles, such as transversus abdominis and internal oblique, both in supine and standing positions. However, the more superficial muscles of the abdominal wall, external oblique and rectus abdominis, did not seem to change in both positions during HE. Previous research studied the effect of HE in abdominal muscles using surface electromyography and demonstrated an activation in the whole abdominal wall [4,5,22]. Moreover, previous investigations analyzed the relationship between the increase of the muscle thickness and the activation with electromyography (EMG,) finding a high correlation between them. However, this technique does not provide information about which muscle was activated, so it is not possible to know whether HE influences each muscle differently, specially according to its depth. Consequently, this study aimed to analyze each muscle separately through ultrasound imaging, which provides information about the changes in their thicknesses. Specifically, our findings suggest an increase in the thickness of transversus abdominis and internal oblique during HE in standing and supine positions, and the external oblique only in standing.

Previously, most studies have focused their research on transversus abdominis since it is the deepest muscle in the abdominal wall and its importance in lumbar stability has been pointed out [23]. The results of this study show that HE increases transversus abdominis’ thickness significantly in comparison with rest, which agrees with Navarro et al. [9], who found an increase of 1.8 ± 1.2 mm during HE in comparison with rest. However, their sample consisted of women with PFM dysfunction and the HE was performed as part of pelvic floor physical therapy, so comparisons should be made carefully since the previous training based on HE in this therapy could improve the muscle activation in comparison with an isolated execution of the HE [24,25].

Transversus abdominis thickness is significantly higher in standing positions in comparison with supine positions. Previous studies reported that only standing is enough to activate transversus abdominis, since it has been described as one of the main muscles needed to maintain an upright position [26,27] and furthermore, it is an important stabilizer of the spine through its insertions in the pelvis, spine and thoracolumbar fascia [23]. These outcomes are consistent with the use of HE as a therapy modality to decrease low back pain, since in most cases a lumbar spine instability is causing the dysfunction and those exercises which improve spine stability find positive results [11,28]. All of these findings, including the fact that in standing position transversus abdominis has significantly more thickness than in supine [15], suggest a pre-activation of the muscle in the upright position and thus, this would be the most appropriate position to work with in HE when the aim is to automatize muscle contraction [29].

Regarding internal oblique, it also increased its thickness during HE in comparison with rest in both positions. The similar results between this muscle and transversus abdominis could be explained by the fact that they do not act as independent muscles, but have a co-activation during contraction and specifically during HE [30]. This co-activation has been described as the abdominal wall muscles being separated but also connected through strong networks of connective tissue [31,32], able to transmit the strength between all the muscular tissue and influence the surrounding muscles [33]. However, there are no differences in the increase of thickness in the internal oblique between the position, so changes are similar in both positions. In this sense, there is a lack of consensus since prior authors have reported that internal oblique is a deep spine stabilizer [34], but others only reported transversus abdominis [35], multifidus [36], diaphragm and PFM [37] as deep stabilizers and conclude internal oblique does not provide stabilization to the spine. There are no significant differences in internal oblique’s thickness between standing and supine position during rest, but there are in transversus abdominis, so findings in this study could support the theory that the internal oblique does not influence the spine stability to the same extent, since it does not increase its thickness significantly during standing as does transversus abdominis [38].

External oblique does not significantly increase its thickness during HE in supine positions, but it does in standing positions, which does not agree with prior research [9] that found a significant increase in external oblique in supine position. These results are controversial since the external oblique’s main function is to approximate the ribs to the middle line [39], and HE requires the expansion and lifting of the rib cage [1,13], but conversely the changes in the thickness of this muscle measured by ultrasound imaging do not agree with its activation [40]. Therefore, external oblique seems to be less involved in spine stabilization, agreeing with previous studies [38].

Finally, the rectus abdominis does not increase its thickness during HE in comparison with rest in any position, which is consistent with other investigations [4,5,9] that support these results, since this muscle is the only vertical in the abdominal wall and the most superficial one, and HE has been described as an exercise for the deeper abdominal muscles, with a higher activation of the longitudinal and oblique fibers [23].

Therefore, this study suggests that HE has more influence the more controlled and deeper the muscles are, strengthening the hypothesis that the increase of the thickness could cause an involuntarily activation during HE due to changes in pressure [1,9]. Furthermore, a voluntary contraction during HE could increase the intraabdominal pressure and damage the PFM [41,42].

In practical terms, this study allows physical therapists and other professionals working with HE a new perspective when addressing an intervention based on these exercises, finding that it is an adequate strategy to improve spine stabilization and all disorders related to it (i.e., low back pain) but other exercises or interventions should be considered if the aim is to influence rectus abdominis or external oblique in particular.

This study has limitations, such as the participants only receiving a familiarization session of HE but they do not perform the exercise usually, so results in subjects who usually perform HE could be different. Besides, only the body mass index was analyzed but body fat, body composition or the level of physical activity of the subjects could also have influenced the results. On the other hand, ultrasonography provides high quality and reliable images of the abdominal wall, but the increase of thickness seems to not be enough to ensure the activation of the muscle, and other reasons should be considered. Previous studies reported a high correlation between the activation and the increase of thickness, measured by electromyography and ultrasound imaging respectively [43,44]. However, combining both techniques probably would provide more consistent results, as well as including other variables of the architectural characteristics of muscles such as cross-sectional area or muscle fascicle length. The levels of activation found by electromyography in prior research and the increase of thickness found in this study are not enough to assume that training based on HE generates an increase of the size of the abdominal muscles [5], so future research could include longitudinal studies and comparison with other abdominal exercises in order to increase the knowledge of the behavior of this area. Finally, these results have been found in healthy participants, but they should be examined carefully when comparing to other populations.

## 5. Conclusions

In conclusion, HE significantly increases the thickness of the transversus abdominis and internal oblique in comparison with rest in both supine and standing positions and also increases the thickness of the external oblique but only in standing positions. These findings should be considered for future interventions with hypopressive exercises in healthy subjects, especially when the aim of the intervention is to improve spine stabilization through the deep abdominal muscles.

## Figures and Tables

**Table 1:** Differences in thickness according to the position and the situation (Mean ± SD).

Muscle (cm)	Supine		Standing	
	Rest (Mean ± SD)	HE (Mean ± SD)	Rest (Mean ± SD)	HE (Mean ± SD)
Transversus abdominis	0.32 ± 0.10 ^#^	0.45 ± 0.18 *^,‡^	0.46 ± 0.13 ^#^	0.58 ± 0.22 *^,‡^
Internal Oblique	0.76 ± 0.27	0.91 ± 0.35 *	0.8 ± 0.34	0.94 ± 0.37 *
External Oblique	0.58 ± 0.18	0.62 ± 0.24	0.56 ± 0.15	0.65 ± 0.23 *
Rectus abdominis	1.03 ± 0.23	1.02 ± 0.2	1.01 ± 0.25	1.01 ± 0.21

* Significant difference (*p* < 0.001) between rest and HE. ^#^ Significant difference (*p* < 0.001) between supine and standing position in rest situation. ^‡^ Significant difference (*p* < 0.001) between supine and standing position in HE situation.
